# Comparison of systemic and mucosal immunization with replicating Single cycle Adenoviruses

**DOI:** 10.15761/GVI.1000128

**Published:** 2018-05-15

**Authors:** William E Matchett, Stephanie S Anguiano-Zarate, Michael A Barry

**Affiliations:** 1 Virology and Gene Therapy Graduate Program, Mayo Clinic, Rochester, MN, USA; 2 Clinical and Translational Science Graduate Program, Department of Internal Medicine, Mayo Clinic, Rochester, MN, USA; 3 Division of Infectious Diseases, Department of Immunology, Mayo Clinic, Rochester, MN, USA

**Keywords:** HIV-1 infections, single immunization, mucosal immunization, Single cycle Adenoviruses

## Abstract

HIV-1 infections occur during sexual contact at mucosal surfaces. Vaccines need to provide mucosal barrier protection and stimulate systemic immune responses to control HIV spread. Most vaccines are delivered by systemic immunization via intramuscular (IM) injection route. While this can drive systemic and mucosal immune responses, there are data show that mucosal immunization may be superior at driving responses at mucosal barriers. To explore this question, we immunized mice with replicating single-cycle adenovirus (SC Ad) vaccines expressing clade B HIV-1 envelope (Env) by intramuscular (IM), intranasal (IN), or intravaginal (IVAG) routes to compare vaccine responses. SC-Ads generated significant antibodies against Env after only a single immunization by the IN route, but not the other routes. These animals were boosted by the same route or by the mucosal IVAG routes. IM and IN primed animals generated strong antibody responses regardless of the boosting route. In contrast, IVAG primed animals failed to generate robust antibodies whether they were boosted by the IVAG or IM routes. These data suggest there may be benefits in first educating the immune system at mucosal sites during HIV vaccination. IN and IM prime-boost were then compared in Syrian hamsters which support SC-Ad DNA replication. In this case, IN immunization again was the only route that generated significant Env antibodies after a single immunization. Following a boost by IN or IM routes, IN primed animals had significantly higher antibody responses than the IM primed animals. Env antibodies could still be detected one year after immunization, but only in animals that received at least one mucosal IN immunization. These data suggest that there is merit in vaccination by mucosal routes.

## Introduction

90% of HIV-1 infections occur at mucosal surfaces [1]. Therefore, it is possible that stopping initial events required for HIV infection may be pivotal for infection prevention [2]. Systemic immunization by the intramuscular (IM) injection can drive mucosal responses against HIV [3-6]. However, other data suggest that mucosal immunization may lead to improved targeting and persistence of immune effectors at mucosal sites (reviewed in [7]).

We previously compared systemic and mucosal immunization using helper-dependent adenovirus (HD-Ad) vectors expressing clade B HIV envelope (Env) in rhesus macaques by the IM or intravaginal (IVAG) route [8]. While IM HD-Ad vaccination generated stronger systemic T cell responses, IVAG immunization generated stronger CD4+ T cell central memory (Tcm) responses in mucosal tissues [8]. When these animals were mucosally challenged by rectal inoculation with SHIV-SF162P3, more animals in the IVAG group resisted infection and had lower viral set points than animals in the IM group [8]. Taken together, these resutls suggested that mucosal immunization might be worthy of additional exploration. Although the IVAG route targets mucosa very close to the rectal challenge site, it is not a feasible vaccination route for use in humans.

In this study, mice and Syrian hamsters were immunized with single-cycle adenovirus (SC-Ad) vectors expressing Env sequences obtained from an HIV-1 patient before and after expansion of antibody neutralization breadth [9]. We compared systemic immunization by the IM route with mucosal immunizations by both the IVAG and the intranasal (IN) routes as well as examined the potential of homologous and heterologous prime-boost immunization strategies for vaccine translation.

## Materials and Methods

### Cell culture

293 cells were purchased from Microbix (Toronto, Ontario, Canada). A549 lung carcinoma were purchased from American Type Culture Collection (ATCC, Manassas, VA). Cells were maintained in Dulbecco’s Modified Eagle Medium with 10% fetal bovine serum (FBS; HyClone, Rockford, IL) and penicillin/streptomycin at 100 U/mL (Invitrogen).

### Adenoviruses

Codon-optimized clade B HIV-1 G4 and F8 envelope sequences [9] were introduced into SC-Ads based on human Ad serotypes 6 as in [10-13]. A control SC-Ad expressing a green fluorescence protein-luciferase (GFP-Luc) fusion protein was also used as a negative control. Viruses were rescued and purified as previously described [10-12].

### Animals

Mice were purchased from (Charles River Laboratories) and Syrian hamsters were purchased from (Harlan Sprague-Dawley). These animals were housed in the Mayo Clinic Animal Facility. Animals were treated in accordance with the policies and procedures of Mayo Clinic’s Institutional Animal Care and Use Committee, the provisions of the Animal Welfare Act, PHS Animal Welfare Policy, and the principles of the NIH Guide for the Care and Use of Laboratory Animals.

### Western Blotting

Human A549 lung cells were plated on 6 well dishes and at the indicated virus particle/cell (vp/cell) ratio with the indicated viruses. 24 hours later, the cells were washed with phosphate-buffered saline (PBS), and the cells were harvested, pelleted, and resuspended in 1X SDS-PAGE loading buffer. Genomic DNA was sheared by sonication and samples were separated on 7.5 to 15% gradient SDS-PAGE Ready Gels (Biorad) prior to western blotting. The blots were incubated with a 1/1000 dilution of H13 anti-HIV Env antibody (NIH AIDS Reagent Program) followed by a 1/10,000 dilution of Protein A/G-HRP (Pierce). Protein bands were detected using Super Signal West Dura Chemiluminescence reagent using an In Vivo F instrument (Kodak).

### Animal immunizations

Mice and hamsters were anesthetized and immunized by the indicated routes using 10^10^ vp per animal. In some cases, the animals were boosted by the same or alternate route with the same dose of the indicated SC-Ad.

### Sample collection

At the indicated times after immunization, animals were anesthetized and blood was collected into BD microtainer tubes with serum separator (Becton Dickinson and Company). Samples were incubated for 1 hour and centrifuged at 13,000×g for 2 min to collect serum.

### Enzyme-linked immunosorbant assay (ELISA)

Immulon 4 HBX plates (Thermo, Milford, MA) were coated with 100 ng/well of SF162 gp140 (NIH AIDS Reagent Program) in 1x PBS and incubated at 4°C overnight. Wells were blocked with 5% milk in Tris-buffered 0.05% Tween-20 (TBST) overnight. Wells were washed and serum samples were added at 1/200 dilutions to plates and incubated at room temperature for 2 hours. Wells were washed, and immunoglobulins were detected with 1/1000 Protein A/G HRP. Wells were washed with TBST and the plates were incubated with 50 μL Ultra TMB ELISA (Thermo Fisher Scientific Inc, Rockford, IL) prior to inactivation with 50 μL 2N H2SO4. OD450 was measured on a Beckman Coulter DTX 880 Multimode Detector system.

### Data Analysis

Statistical analyses were performed using Prism Graphical and JMP software.

## Results

### SC-Ad Expressing HIV-1 gp160

Most published gene-based Adenovirus (Ad) vaccines are replication-defective Ad (RD-Ad) vectors with their E1 gene deleted. A RD-Ad delivers its single copy of antigen gene and expresses “1X” of this protein. In contrast, an E1 intact replication-competent Ad (RC-Ad) delivers one copy, but then replicates the antigen gene DNA 10,000-fold to amplify antigen production and immune responses [14-25]. Although RC-Ads are consistently reported to be substantially more potent than RD-Ads, RC-Ads actually risk causing adenovirus infections in patients (reviewed in [26]).

**Figure 1.**
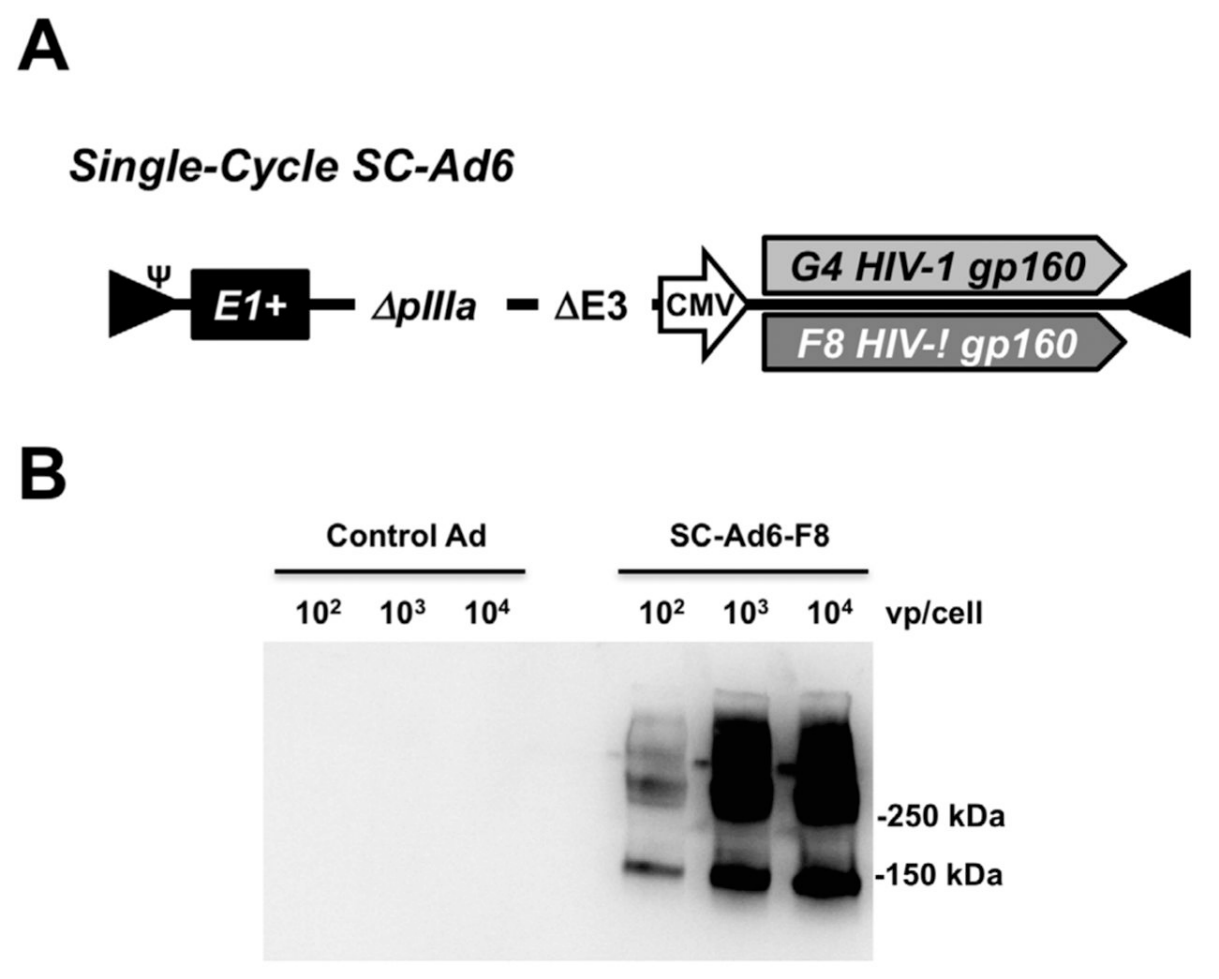
SC-Ad Vectors Expressing HIV-1 Envelope. **(A)** Diagram of SC-Ad expressing either the G4 or F8 clade B gp160 envelope gene. E1+ indicates the presence of the Ad E1 gene to facilitate DNA replication. DpIIIA indicates deletion of this late Ad gene to disrupt the production of infectious progeny viruses. (**B)** Western blot detecting expression of F8 gp160 in cells infected with the indicated amounts of the indicated SC-Ad in vp/cell. The control SC-Ad expresses GFP-Luc.

To take advantage of DNA replication by Ads, but avoid the risk of adenovirus infection, we developed that retain their E1 genes, but that are deleted for their pIIIa gene to block the production of infectious progeny virions (Fig. 1A and [10– 12, 26, 27]). SC-Ads replicate their genomes and transgenes as well as RC-Ad (up to 10,000-fold) [10], but actually generate more robust and persistent immune responses than either RD-Ad or RC-Ad [12].

Clade B envelope sequences were isolated from an HIV-1 patient before and after their antibody responses underwent expansion of HIV neutralization breadth (G4 and F8 gp160 sequences, respectively) [9]. These Env sequences were inserted into SC-Ad based on lower seroprevalence human adenovirus serotype 6 (Figure 1A). G4 and F8 SCAd6’ s were rescued and produced in 293-IIIA cells and purified on CsCl gradients as in [10,12,26,27]. When used to infect A549 cells, both vectors produced gp160 as determined by western blotting (Figure 1B and data not shown).

### Mucosal and systemic immunization in small animals

**Figure 2.**
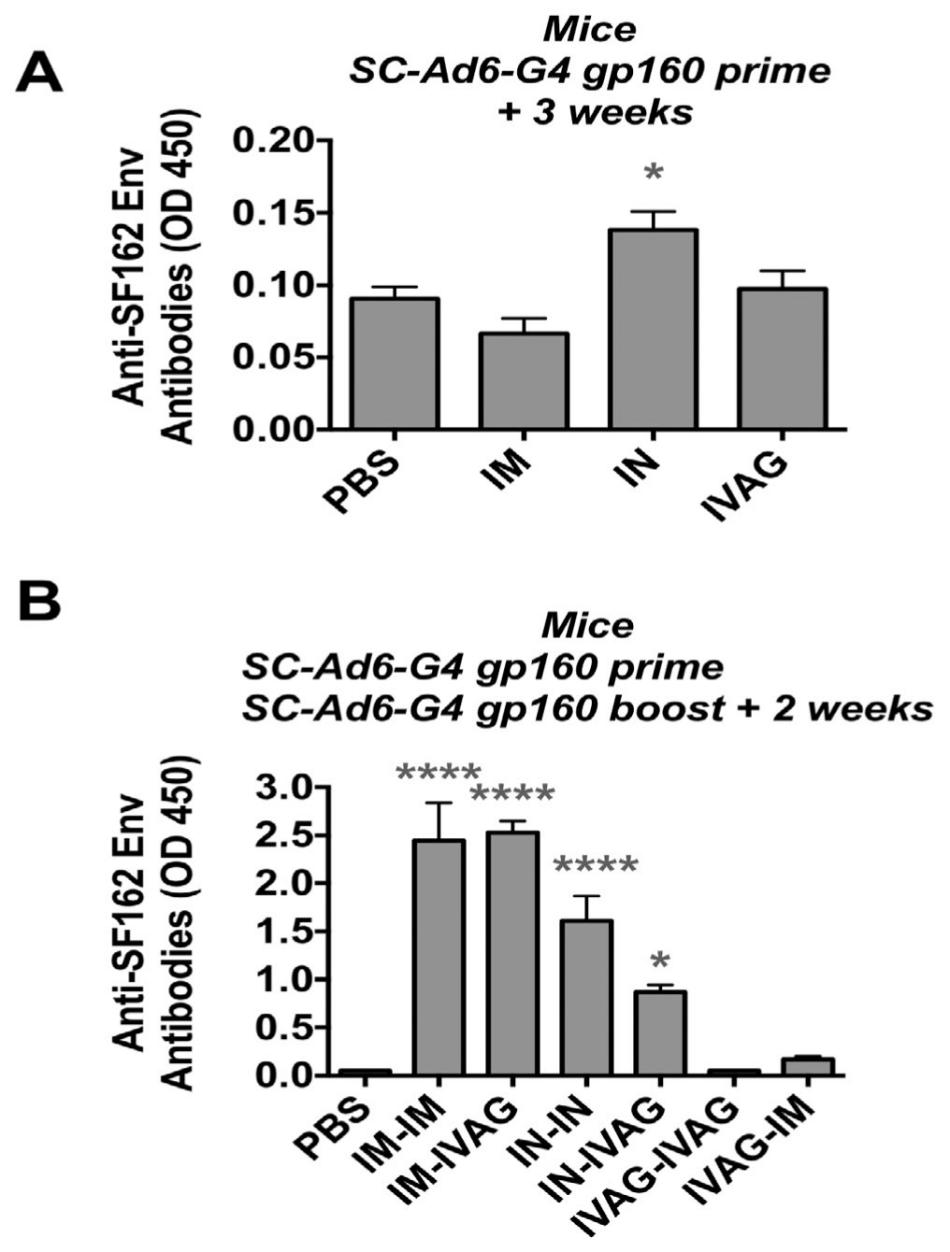
Antibodies Against HIV-1 Env in Mice. Mice were immunized with 1010 vp of the indicated SC-Ad by the indicated route and ELISAs were performed after priming or boosting. (A) Anti-Env antibodies after one immunization. (B) Anti-Env antibodies after prime-boost immunization. * p < 0.05, **** p < 0.0001 by one way ANOVA.

We previously tested Highly dependent vectors in small animals and rhesus macaques by the systemic IM route and by a variety of mucosal routes including oral gavage, oral enteric coated capsules, IN, and IVAG [8,10-12,26-30]. To test the clade B expressing SC-Ads by different routes, SCAd6- G4 was administered via IM, IN, and IVAG immunizations (Figure 2). ELISA against SF162P3 Env demonstrated that only the IN immunized group generated significant anti-Env antibodies after single immunization (Figure 2A, p < 0.05 by ANOVA). The mice were boosted with the same SC-Ad6-G4 vector by the same route or an opposite route and antibodies were measured 2 weeks later (Figure 2B). Under these conditions, the IM-IM and IM-IVAG immunized mice had the highest antibody levels and IN-IN and IN-IVAG animal antibodies were lower, but still significantly different than the PBS group (p < 0.001 for IN-IN, p < 0.05 for the IN-IVAG group). Neither of the IVAG-IVAG or I VAGIN immunized groups generated significant antibody responses.

**Figure 3.**
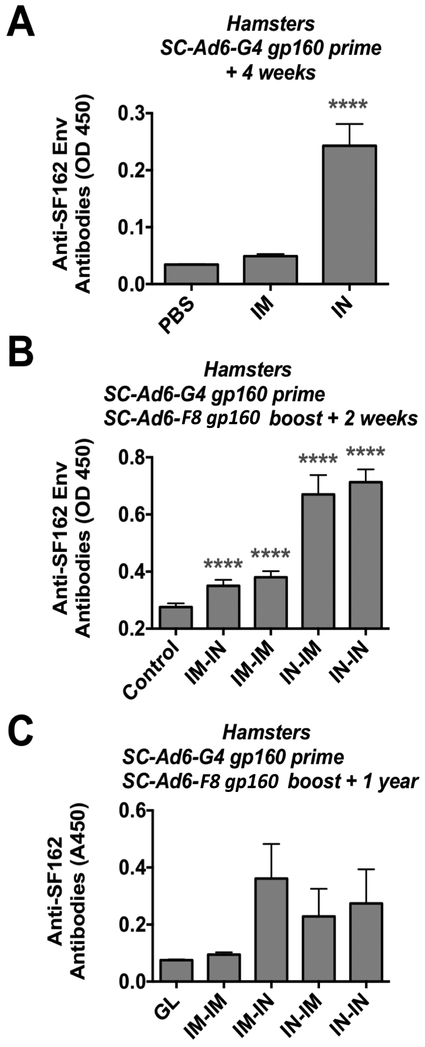
Antibodies Against HIV-1 Env in Syrian Hamsters. Hamsters were immunized with 1010 vp of the indicated SC-Ad by the indicated route and ELISAs were performed after priming or boosting. (**A)** Anti-Env antibodies after one immunization. (**B)** Anti-Env antibodies after prime-boost immunization. (**C)** Anti-Env antibodies 1 year after last immunization.

Mice do not support Ad DNA replication well, resulting in the underestimation of SC-Ad potency. In contrast, Syrian hamsters support the full Ad viral cycle, allowing single cycle DNA replication to occur [11,12,27,31,32]. Given the weak IVAG responses in mice, this route was omitted and hamsters were primed with SC-Ad6-G4 by the IM and IN routes. ELISA data 4 weeks after priming demonstrated significantly higher anti-Env responses in the IN group when compared to PBS or IM groups (Figure 3A, p < 0.001 by ANOVA). The animals were then boosted with SC-Ad6-F8 by the same or opposite IM or IN routes and antibodies were measured 2 weeks later. Under these conditions, all immunized animals had significantly higher antibodies than PBS controls (Figure 3B, p < 0.001), but the IN-IN and IN-IM animals were significantly higher than all other groups.

These hamsters were held for 1 year after last immunization and tested by ELISA. This showed the residual presence of anti-Env antibodies in the IM-IN, IN-IM, and IN-IN groups, but not in the IM-IM group (Figure 3C).

## Discussion

We have previously compared systemic and mucosal vaccination by a number of routes in small animals and in rhesus macaques. In macaques, animals were pre-immunized with Ad serotype 5 (Ad5) and then vaccinated four times by the IM or IVAG routes by serotype switching with four species C HD-Ads: Ad6, Adi, Ad5, and Ad2. No protein boosts were applied. The animals were then challenged with SHIV-SF162PE by the mucosal rectal route [8]. 75% of IVAG animals had viral set points near the limits of detection, whereas only 25% of IM immunized animals reached this low level of viremia.

This finding ran counter to immune correlates collected from plasma and PBMCs. Virtually all humoral and cellular immune responses tested from the blood were stronger in the IM group. For example, the IM route generated markedly stronger T effector memory (Tern) and T central memory (Tcm) cells in PBMCs than the IVAG route. In contrast, when responses were measured from colon biopsies, CD8+ responses were stronger in the IVAG group. All animals in the IVAG group generated CD4+ Tcm cells in the colon. Three of the four animals from the IM group generated Tcm cells, but these were at lower levels than in the IVAG animals.

These data suggested that mucosal immunization does indeed have advantages when vaccinating against the mucosal pathogen HIV-1. However, the IVAG route is not likely to be easily used in humans. In this study, we tested different routes of immunization with newer gene replicating SC-Ad vectors in small animals as a prelude to testing in more expensive rhesus macaque models. We show that the IN route is superior to IVAG in small animals and that vaccination by that route can generate significantly higher antibody responses after a single immunization when compared to the traditional IM route. While the IN route was robust for priming in mice, IM primed animals ultimately generated higher anti-Env antibodies after boosting by IM or IVAG routes than IN primed animals. In hamsters, IN primed animals generated stronger Env antibodies after IN or IM boosting. This difference in the two species may reflect the ability of SC-Ad to replicate its DNA in hamsters and its inability to strongly replicate in mice. This may also reflect better SC-Ad DNA replication in epithelial cells in the nares of animals when compared to weak replication in terminally differentiated myotubes in the muscle.

These data suggest that the IN route is superior to the previously tested IVAG route for SC-Ad vectors. These data lay the foundation for exploring the effects of systemic and mucosal vaccination on protection against mucosal pathogens like HIV-1.
